# Nano-emulsion of mangosteen rind extract in a mucoadhesive patch for periodontitis regenerative treatment: An in vivo study

**DOI:** 10.1016/j.jtumed.2022.03.003

**Published:** 2022-03-23

**Authors:** Mohammed A. Aljuanid, Huda R. Qaid, Dur M. Lashari, Rini D. Ridwan, Hendrik S. Budi, Baleegh A. Alkadasi, Yeka Ramadhani, Riski Rahmasari

**Affiliations:** a Doctoral Program of Dental Medicine, Faculty of Dental Medicine, Universitas Airlangga, Surabaya, Indonesia; b Department of Oral and Dental Medicine, Faculty of Medicine, Taiz University, Taiz, Yemen; c Faculty of Oral and Dental Medicine, Alsaeed University, Taiz, Yemen; d Department of Oral Biology, Faculty of Dental Medicine, Universitas Airlangga, Surabaya, Indonesia; e Head of Oral Medicine and Periodontology Department, Faculty of Dentistry, Ibb University, Yemen; f Undergraduate Program of Dental Medicine, Universitas Airlangga, Surabaya, Indonesia

**Keywords:** مستخلص قشرة مانغوستين, رقعة اللثة اللاصقة المخاطية, مستحلب النانو, التهاب اللثة, بكتيريا البورفيروموناس, Mangosteen rind extract, Mucoadhesive gingival patch, Nano-emulsion, *P. gingivalis*bacteria, Periodontitis

## Abstract

**Objective:**

To investigate the therapeutic potential of nano-emulsion of mangosteen rind extract in a mucoadhesive gingival patch on periodontitis, and its effect on tumor necrosis factor alpha (TNF-α), receptor activator of nuclear factor kappa Β ligand (RANKL), and interleukin 10 (IL-10) expression.

**Methods:**

Sixty Wistar rats were divided into four groups: positive control group (mucoadhesive patch with doxycycline), negative control group (mucoadhesive patch), treatment group I (mucoadhesive patch with mangosteen rind extract), and treatment group II (mucoadhesive patch with nano-emulsion of mangosteen rind extract). An experimental model of *Porphyromonas gingivalis*-induced periodontitis was established in rats by treatment with 0.03 mL bacteria locally (1 × 10^10^ colony-forming units) seven times at 2-day intervals in the gingival sulcus of mandibular anterior teeth. Treatment was 1 h/day for 3 days. On days 3, 5, and 7, five rats from each group were killed. TNF-α, IL-10, and RANKL expression was determined by dissecting the lower jaw for immunohistochemistry.

**Results:**

The mucoadhesive patch with nano-emulsion mangosteen rind extract significantly decreased TNF-α and RANKL expression and increased IL-10 expression (p < 0.05) compared to the treatment I, positive and negative control groups.

**Conclusion:**

A mucoadhesive gingival patch with nano-emulsion of mangosteen rind extract has the potential to treat periodontitis by decreasing TNF-α, RANKL, and increasing IL-10 expression.

## الملخص

**أهداف البحث:**

دراسة الإمكانات العلاجية لمستحلب النانو لمستخلص قشرة مانغوستين في رقعة لاصقة مخاطية على التهاب اللثة، وتأثيرها على عامل نخر الورم ألفا، ولجين منشط مستقبلات العامل النووي كابا ب، وتعبير إنترلوكين-10.

**طرق البحث:**

تم تقسيم ستين فأرا إلى أربع مجموعات: مجموعة التحكم الإيجابي (رقعة اللاصق المخاطي مع الدوكسيسيكلين) ، والتحكم السلبي (رقعة اللاصق المخاطي) ، والمجموعة العلاجية الأولى (رقعة اللاصق المخاطي مع خلاصة قشرة المانجوستين) ، والمجموعة العلاجية الثانية (رقعة اللاصق المخاطي مع مستحلب النانو من مستخلص قشر مانغوستين). تم إنشاء نموذج تجريبي لالتهاب دواعم السن الناجم عن البورفيروموناس في الجرذان عن طريق العلاج بـ 0.03 مل من البكتيريا محليا سبع مرات بفاصل يومين في التلم اللثوي للأسنان الأمامية للفك السفلي. كان تطبيق العلاج لمدة ساعة / يوم لمدة 3 أيام. في الأيام 3 و 5 و 7 بعد العلاج، تم التضحية بخمسة فئران من كل مجموعة. تم تحديد عامل نخر الورم ألفا، ولجين منشط مستقبلات العامل النووي كابا ب، وتعبير إنترلوكين-10، عن طريق تشريح الفك السفلي للكيمياء المناعية.

**النتائج:**

أدت الرقعة اللاصقة المخاطية مع مستخلص قشرة مانغوستين لمستحلب النانو إلى انخفاض كبير في تعبير عامل نخر الورم ألفا، ولجين منشط مستقبلات العامل النووي كابا ب، وزيادة تعبير إنترلوكين-10. مقارنة بالمجموعة العلاجية الأولى ومجموعات التحكم الإيجابية والسلبية.

**الاستنتاجات:**

إن الرقعة اللاصقة المخاطية مع مستحلب نانو من مستخلص قشرة المانجوستين لديها القدرة على علاج التهاب اللثة عن طريق تقليل تعبير عامل نخر الورم ألفا، ولجين منشط مستقبلات العامل النووي كابا ب، وزيادة تعبير إنترلوكين-10.

## Introduction

Periodontitis is a common inflammatory oral disease that exists on a wide scale worldwide and is the main cause of tooth loss.^1^ Periodontitis affects the periodontal tissues with a moderate to slow rate of progression.^2^ The recent Global Burden of Disease Study shows that periodontitis is the sixth most prevalent disease worldwide, with an overall prevalence of up to 20%.^3^ Periodontitis, which is caused by long-lasting bacterial plaque and resultant calculus accumulation, is considered the primary etiologic factor in initiating periodontitis.^4^^,^^5^ *Porphyromonas gingivalis* is the most important periodontal pathogen and mediates periodontal disease. The host's inflammatory responses result in edema and infiltration of leukocytes, which release inflammatory mediators, eventually leading to progressive damage to the periodontal ligament and alveolar bone, together with gingival recession, pocket depth formation, or both. Therefore, inflammatory mediators play a significant role in periodontal disease onset and progression. In addition, the amount of inflammatory mediators is linked to periodontal disease.^4^, ^5^, ^6^

Interleukin-1β (IL-1β), IL-6, tumor necrosis factor alpha (TNF-α), and receptor activator of nuclear factor kappa B ligand (RANKL) are significant markers of the pathogenesis of periodontitis.^7^ *P. gingivalis* infection successfully induces the loss of alveolar bone, significantly activates osteoclasts, and increases the expression of IL-17, IL-1β, and RANKL in periodontitis.^8^ TNF-α is a major pro-inflammatory cytokine involved in the inflammatory phase of periodontitis, and is considered a major contributor to bone pathophysiology due to its stimulation of bone resorption. IL-6 and TNF-α induce the osteoclastic pathway through the expression of RANKL by osteoblasts and TNF-α via macrophage colony-stimulating factor (M-CSF) for the activation of preosteoclasts into osteoclasts.^9^ Moreover, RANKL is an important factor for osteoclastogenesis and is expressed by osteoblasts, osteocytes, and stromal cells. RANKL binds to its receptor on RANK, which is expressed by osteoclast precursor cells, causing the activation of osteoclast cells and resulting in the process of osteoclastogenesis.^10^ Therefore, the increase in RANKL plays a key role in periodontitis bone loss.^8^ On the other hand, the anti-inflammatory cytokine IL-10 plays an important role in suppressing the progress of periodontitis and regulating bone metabolism. A previous study showed that IL-10 knockout mice are highly susceptible to *P. gingivalis*-induced periodontitis. IL-10 also inhibits neutrophil migration.^11^ The secretion of IL-10 by regulatory T lymphocytes can suppress osteoclastogenesis by inhibiting the function of the T helper 1 cell (Th1) effector cytokine IL-6. IL-10 can also suppress RANKL by stimulating an increase in osteoprotegerin (OPG). Consequently, IL-10 can inhibit osteoclast differentiation and maturation by regulating OPG secretion and decreasing RANKL expression and M-CSF.^12^

Plants and herbs have been used for medicinal purposes for decades to treat many diseases. Natural sources for preventing immunological complications have long garnered the attention of researchers. The queen of fruits *Garcinia mangostana* Linn (mangosteen) is one of the sources of plant-derived medicines. It typically originates in tropical Southeast Asian countries such as Malaysia, Indonesia, and Thailand.^13^ Mangosteen rind consists of active chemical ingredients such as xanthone, flavonoid, saponin, tannin, phenol, gartanine, garcinon, vitamins B1, B2, terpenes, anthocyanins, and other biologically active substances that support its medicinal properties.^14^ The significant bioactive secondary metabolites of mangosteen are xanthones derivatives (i.e., α- and γ-mangosteen have antioxidant, antiproliferative, antibacterial, and anti-inﬂammatory eﬀects).^15^^,^^16^ Xanthone substances have considerable ability to regulate and minimize oxidative damage by hampering or preventing oxidation caused by reactive oxygen species, thus inhibiting the degeneration of cells. However, they also stimulate the regeneration of body cells, which break down quickly.^17^ Similarly, they suppress inﬂammatory processes by blocking the production of cyclooxygenase (COX) and lipoxygenase (LOX), immediately inhibiting the activity of I kappa B (IκB) and COX2 gene transcription (nuclear factor kappa B [NF-κB] target gene). The inhibition of COX and LOX enzymes result in the release of prostaglandins, prostacyclins, thromboxanes, and leukotrienes, which suppress inﬂammatory processes, marked by a reduction in inﬂammatory cell number.^18^ Moreover, a study in U037 macrophage-like cells revealed that the release of inflammatory markers TNF-α and IL-4 can be inhibited by 20 μg/mL α-mangostin.^19^

Nano-based drug delivery systems offer new opportunities for effective and specified oral disease treatment. Nano-emulsion is an advanced nanotechnology method used to deliver and enhance the bioavailability of the medication and therapeutic agent. It can also be used as a delivery system for herbal therapeutic agents in diverse applications.^20^ Nano-emulsion is defined as a lipid-based thermodynamically stable drug delivery system whose particles are measured on a nanometer scale. It is an optically transparent system made up of a surfactant (i.e., oil, cosurfactant, nanometer-sized water droplet).^1^ Nano-emulsions have broad spectrum antimicrobial activity due to their ability to fuse with and lyse these microorganisms.^21^ Nano-emulsion can be used for periodontal disease treatment as it is safe, non-irritating to mucous membranes, and highly effective against periodontopathic bacteria. Nanodroplets and surfactants react with the outer membrane of bacteria, breaking it down and killing the bacteria.^22^ In addition, nano-emulsion enhances drug solubility, promotes periodontal mucosa permeability, and delivers the drug dose with fewer side effects that result in more effectiveness of the drug.^1^ The mechanical and drug–release properties of the nano-formulation are some of the most significant factors in developing the clinical efficacy of treating patients. As a result, an optimum nano-formulation should be developed so that it can be delivered to a specific site with controlled drug release and more gingival mucosa retention for a definite period.

A mucoadhesive drug delivery system is a safe delivery system compared to other drug administration routes. It has several advantages over conventional administration methods, such as the ability to control the release of drug dosage at specific sites with the extended retention time of drugs at target sites. One of the most significant advantages of these systems is avoidance of the first phase of metabolism.^23^ Hence, this study investigated the therapeutic potential of nano-emulsion of mangosteen rind extract in a mucoadhesive gingival patch on a periodontitis model induced by *P. gingivalis* and its effect on TNF-α, IL-10, and RANKL expression.

## Materials and Methods

### Mangosteen rind extraction

**Figure 1:**
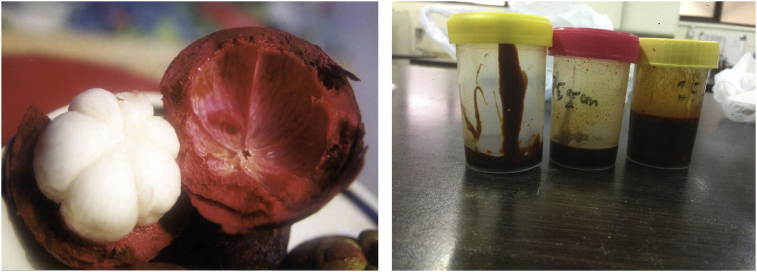
Mangosteen white fleshy fruit covered with red pericarp (rind)^26^ (left panel); mangosteen rind extract (right panel).

The fruits of mangosteen rind were obtained from Blitar City East Java Indonesia (Figure 1). The rinds were cleaned under running water, chopped into small pieces, and then placed in a hot oven to dry for 72 h at 50 °C. The samples were sieved after grinding them into a fine powder (20 mesh). The dried powder of mangosteen rind was extracted using the maceration technique with 96% ethanol at room temperature (25–28 °C). Then the resultant extract was filtered using filter paper (Whatman No. 1) and evaporated to dryness using a rotary vacuum evaporator at 50 °C (G3; Heidolph Instruments, Schwabach, Germany) (Figure 1).^24^^,^^25^

### Preparation of the nano-emulsion of mangosteen rind extract

A high-speed homogenization process with a magnetic stirrer was used to create a nano-emulsion for mangosteen extraction. The oil phase consisted of virgin coconut oil, mangosteen rind extract, and surfactants. The surfactants (i.e., Span 80 and Tween 80) were mixed to create a hydrophile-lipophile balance. Preparation of the oil phase was done by mixing coconut oil (41.7 g) with Tween (29.16 g) and Span (29.16 g) surfactants at a ratio 1:1.4 (v.v) using a magnetic stirrer at 500 rpm for 10 min. The nano-emulsions were created by adding the oil phase to distilled water of a ratio of 1:1.4 (v/v), followed by stirring with a magnetic stirrer at 750 rpm until thoroughly mixed. Mangosteen rind extract (4 g) was a mixture and stirred continuously with a magnetic stirrer at 44 °C and 8000 rpm for 15 min. Finally, a nano-emulsion at a concentration of 4% was formed.^27^^,^^28^

### Characterization of nano-emulsion droplets

The nano-emulsion was characterized by determining the droplet size, zeta potential, and polydispersity index (PDI) using the Zetasizer® Nano ZS apparatus (Malvern Instruments Ltd., Malvern, UK). The nano-emulsion was dispersed into the disposable sizing cuvette cell of the device. Photon correlation spectroscopy was used to quantify the hydrodynamic diameter and PDI of nano-emulsion droplets at 20 °C and a scattering angle of 173°. The Malvern Zetasizer equipment measures the variation in light scattering as a function of time due to Brownian particle motion. At 25 °C, the zeta potential was measured using Zetasizer® Nano ZS equipment (Malvern Instruments).^27^^,^^29^

### Preparation of the mucoadhesive gingival patch

Mucoadhesive gingival patches were prepared using solvent evaporation (solvent casting technique). Initially, the ionic polymer sodium carboxymethyl cellulose (CMC-Na) (Teknis Indonesia) was used to prepare the patches. To improve the patch's performance and release characteristics, propylene glycol (PG) was utilized as a plasticizer. To improve the solubility of the polymer, 0.6 g CMC-Na was dispersed in 30 mL warm distilled water. Then, under continuous stirring, PG (1 g) plasticizer was added to achieve sufficient viscosity dispersion. Then the solution was poured into Petri dishes, kept at 4 °C for 2 days to eliminate any trapped air bubbles, and oven-dried for 96 h at 30 °C. All of the patches were uniform, homogeneous, and bubble-free. The patch had a final thickness of 0.3 mm and a diameter of 0.3 mm.^30^, ^31^, ^32^, ^33^

### Preparation of a mucoadhesive gingival patch with nano-emulsion of mangosteen rind/mangosteen rind/doxycycline antibiotic

The procedure involved adding 0.6 g CMC-Na into 30 mL warm water followed by manual stirring to increase the solubility of the polymer. Then mangosteen rind extracts 40 g (4%), nano-emulsion of mangosteen rind extract 40 g (4%), and doxycycline 0.7% (Kimia Farma, Jakarta, Indonesia) were added separately to the previous mixture and stirred until it became a homogeneous mixture. Subsequently, PG (1 g) plasticizer was added with continuous stirring until we obtained a suitable viscosity dispersion. The mixtures were poured into Petri dishes and dried in the oven at 50 °C for 168 h. All patches were uniform, homogeneous, and free from bubbles. The final thickness of the patch was 0.3 mm in diameter.^31^^,^^32^

### Experimental model

The Laboratory of the Faculty of Veterinary Medicine, Airlangga University approved the study protocol. This was an experimental laboratory study with a post-test-only control group design, which used male Wistar rats (*Rattus novergicus*). Rats were 5–6 months old and weighed 250–300 g. According to Lemeshow,^34^ a sample size of 60 models was needed, with each group having five animals for a total of 12 groups.

### Induction of model periodontitis and treatment application

The rats were acclimatized for 1 week and then induced by 0.03 mL *P. gingivalis* bacteria locally (1 × 10^10^) colony-forming units seven times at 2-day intervals in the mandibular gingival incisive sulcus of the anterior teeth of rats.^35^, ^36^, ^37^ Then rats with reported periodontitis (i.e., swelling and resorption between teeth) were anesthetized with 0.1 mL/100 g body weight of ketamine intramuscularly. The gingival patches were cut and sized into small pieces to fit the incisive gingival mucosa of the rat's jaw and then placed using dental tweezers. The mucoadhesive gingival patch containing 0.7% doxycycline antibiotic for the positive control group, 4% mangosteen rind extract for treatment group I, and nano-emulsion of 4% mangosteen rind extract for treatment group II was applied and retained in the periodontitis area of the rats for 1 h/day for 3 days.^38^^,^^39^ Then, all animals were killed with ketamine injection (0.4 mL/100 g) on days 3, 5, and 7 after treatment, followed by biopsy of the anterior region of the mandible. The animals were buried according to the ethics of experimental animals.^40^

### Immunohistochemistry

After the rats were killed, indirect immunohistochemistry (IHC) was performed on the anterior mandibular of rats.^41^ The expression of TNF-α, RANKL, and IL-10 was determined by counting the number of macrophages and osteoblasts that were immunoreactive with monoclonal anti-TNF-α, anti-IL-10, and anti-RANK antibodies under a light microscope (H600L; Nikon, Tokyo, Japan) at 400 × magnification in five fields of view.^42^

### Statistical analyses

Data analyses were conducted with SPSS version 25, and the data are presented as the mean ± standard deviation (SD). The Shapiro–Wilk normality test was used, followed by one-way analysis of variance and Tukey's post hoc test. A statistically significant difference was defined as p < 0.05.

## Results

### Nano-emulsion

Determination of the nano-emulsion for its important characteristics was recorded five times as shown in Table 1. The data on mangosteen rind extract nano-emulsion droplet size, PDI, and zeta potential are expressed as the mean ± SD values (Table 1)**.** The mean droplet size (diameter) of the nano-emulsion was 335.68 ± 62.991 nm, the PDI was 0.4038 ± 0.128, and the zeta potential was −11.966 ± 10.702 mV.

**Table 1:** Determination of droplet size, PDI, and zeta potential of mangosteen rind extract nano-emulsion.

Characteristic	Record 1	Record 2	Record 3	Record 4	Record 5	Mean ± SD
Droplet Size (d. nm)	406.1	355.2	376.6	258.1	282.4	335.68 ± 62.991
PDI	0.317	0.362	0.325	0.629	0.386	0.4038 ± 0.128
Zeta Potential (mV)	−5.93	−7.63	−7.07	−20.5	−25.7	−11.966 ± 10.702

### Immunohistochemical staining results of periodontal tissues of rats in each group

TNF-α, RANKL, and IL-10 expression, in response to administration of the mucoadhesive gingival patch for the treatment I, treatment II, positive control, and negative control groups, was determined by IHC of the mandibular preparation of the anterior incisive region.

### TNF-α expression

**Figure 2:**
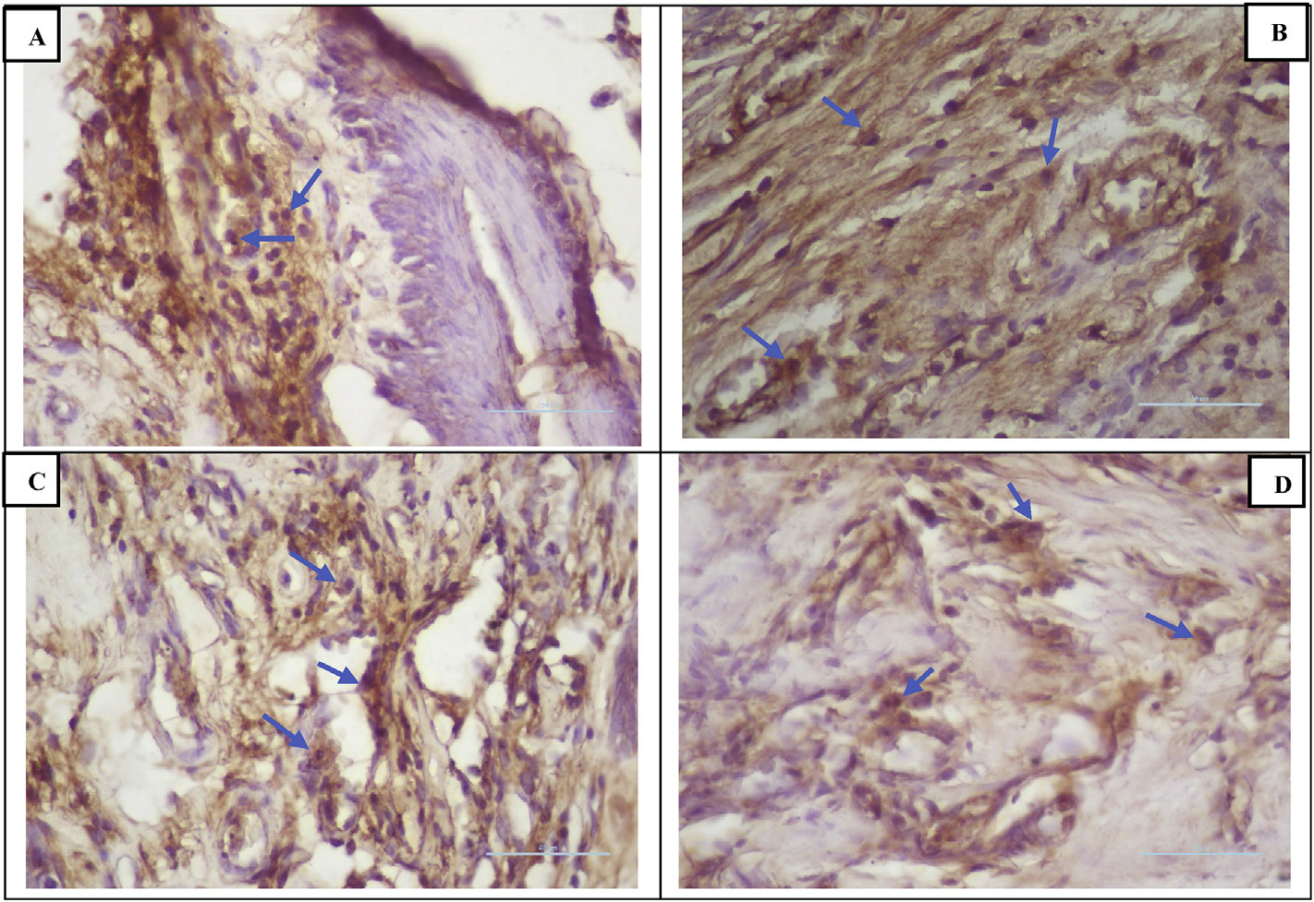
Immunohistochemistry of TNF-α expression (blue arrow). (A) Negative control group; (B) positive control group; (C) treatment I group; (D) treatment II group (400 × magnification).

**Graph 1:**
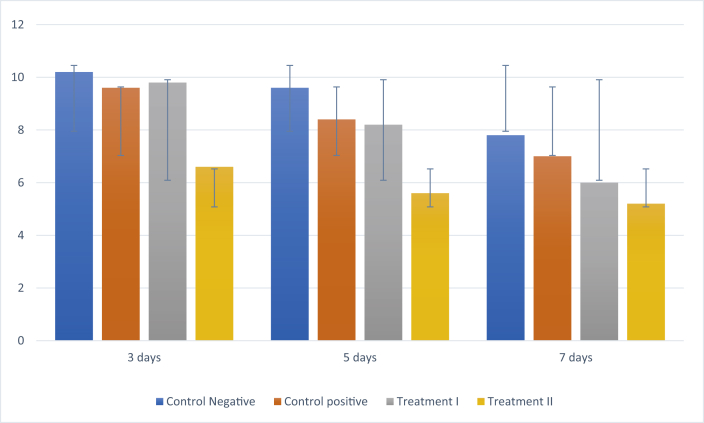
Mean ± SD of the level of TNF-α expression.

IHC analyses showed a reduction in TNF-α expression in all groups following treatment on days 3, 5, and 7 (Figure 2). Treatment group II had the lowest TNF-α expression compared to the other groups (Graph 1)**.** The results of Tukey's post-hoc test showed that TNF-α expression was significantly different between the negative control group and treatment II group after 3, 5, and 7 days (p < 0.001, p < 0.001, and p < 0.001, respectively) and between the negative control group and treatment I group after 7 days (p < 0.041). However, there was no significant difference in expression between the negative and positive control groups after 3, 5, and 7 days (p < 0.494, p < 0.915, and p < 0.915, respectively) or between the negative control group and treatment I group after 3 and 5 days (p < 0.746, and p < 0.724 respectively).

### RANKL expression

**Figure 3:**
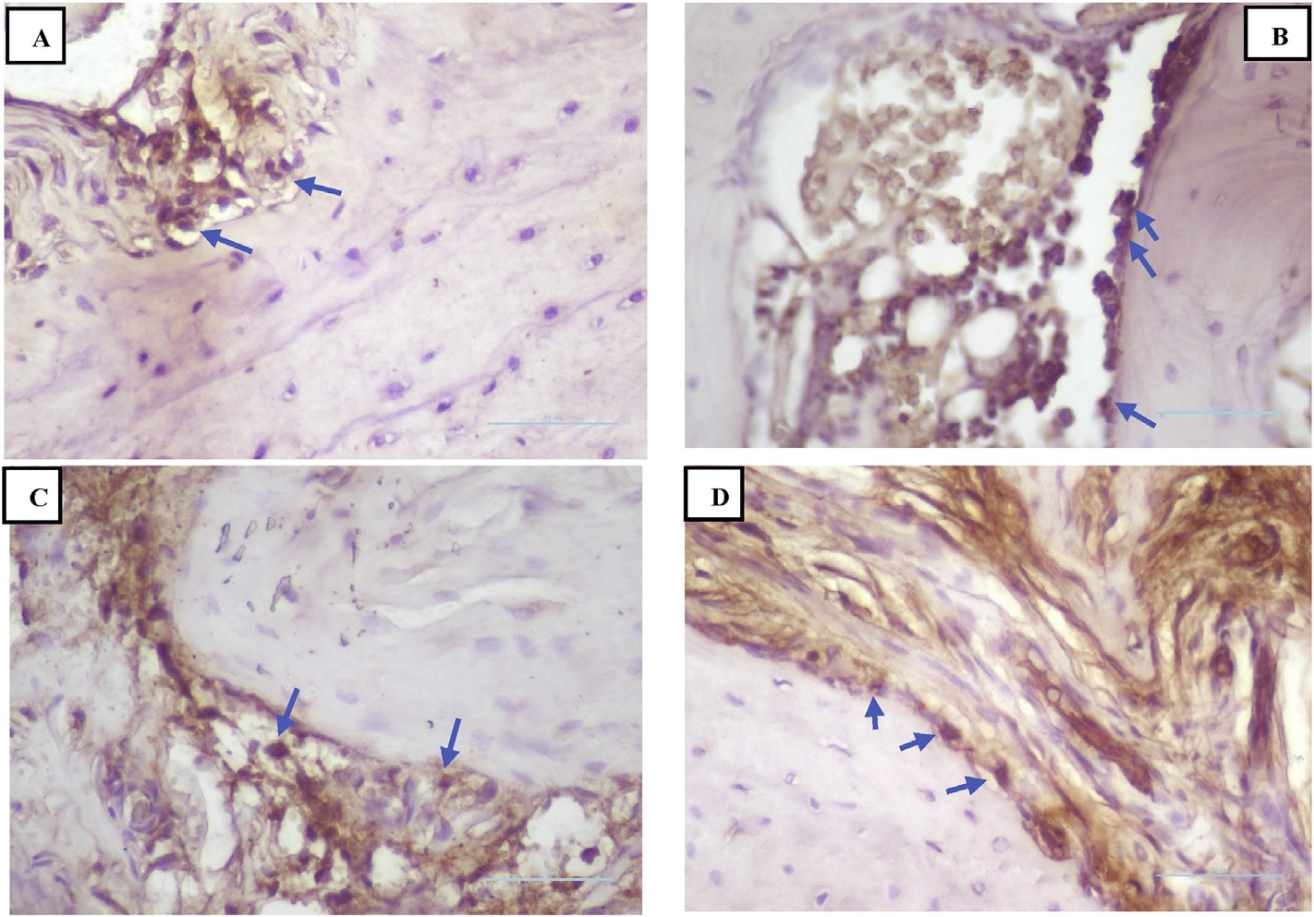
Immunohistochemistry of RANKL expression (blue arrow). (A) Negative control group; (B) positive control group; (C) treatment I group; (D) treatment II group (400 × magnification).

**Graph 2:**
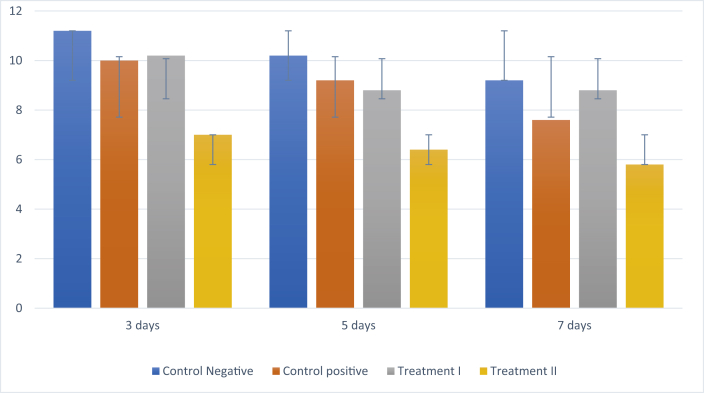
Mean ± SD for calculating the level of RANKL expression.

IHC analyses showed that there was a reduction in RANKL expression in all of the groups following treatment on days 3, 5, and 7 (Figure 3). Treatment group II had the lowest RANKL expression on the three interval days compared to the other groups (Graph 2)**.** The results of Tukey's post hoc test showed that RANKL expression was significantly different between the negative control group and treatment II group after 3, 5, and 7 days (p < 0.001, p < 0.001, and p < 0.001, respectively). However, there was no significant difference in expression between the negative and positive control groups after 3, 5, and 7 days (p < 0.494, p < 0.764, and p < 0.123, respectively) or between the negative control group and treatment I group after 3, 5 and 7 days (p < 0.746, p < 0.268, and p < 1.000, respectively).

### IL-10 expression

**Figure 4:**
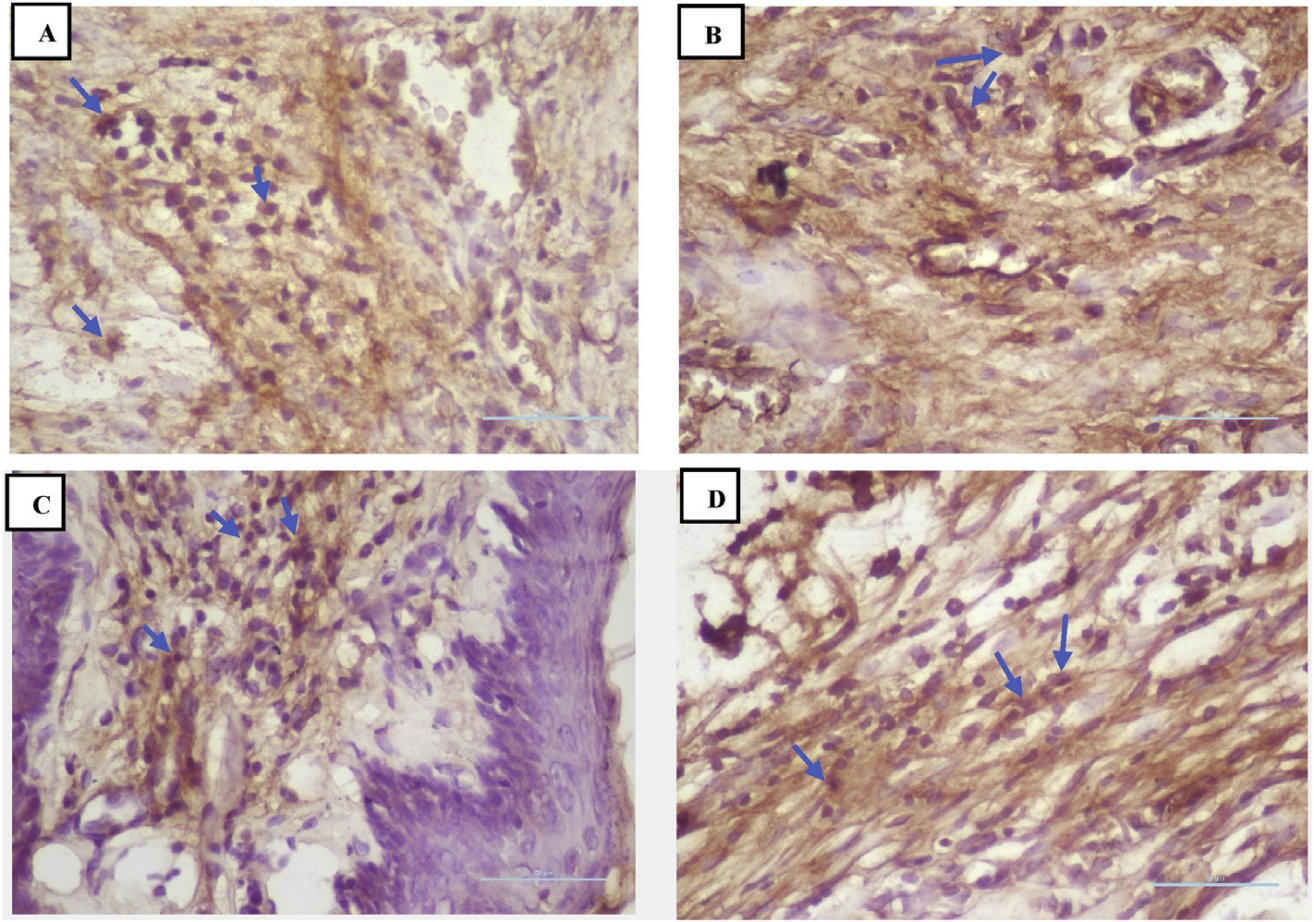
Immunohistochemistry of IL-10 expression (blue arrow). (A) Negative control group; (B) positive control group; (C) treatment I group; (D) treatment II group (400 × magnification).

**Graph 3:**
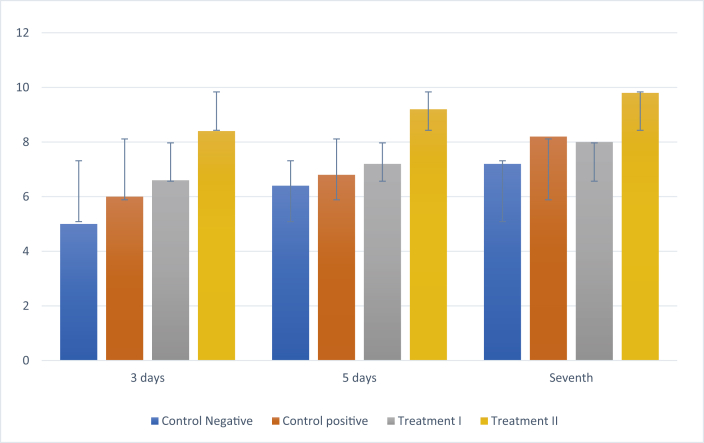
Mean ± SD of the level of IL-10 expression.

There was increase in IL-10 expression in all groups after treatment on days 3, 5, and 7, whereas the negative control group had the lowest IL-10 expression (Figure 4). Treatment group II showed the highest IL-10 expression on days 3, 5, and 7 compared to the other groups (Graph 3)**.** The results of Tukey's post hoc test showed that IL-10 expression was significantly different between the negative control group and treatment II group after 3, 5, and 7 days (p < 0.001, p < 0.001, and p < 0.001, respectively). However, there was no significant difference in expression between the negative and positive control groups after 3, 5, and 7 days (p < 0.604, p < 0.999, and p < 0.604, respectively) or between the negative control and treatment I group after 3, 5 and 7 days (p < 0.053, p < 0.857, and p < 0.915, respectively).

## Discussion

Due to its antimicrobial activity, mangosteen rinds can reduce the formation of plaque and calculus since they are responsible for many common oral diseases, including gingivitis, periodontitis, dental caries, and peri-implantitis. According to some research, mouthwash containing mangosteen pericarp has benefits as a periodontal treatment and for controlling oral malodor.^43^

IL-6, TNF-α, prostaglandin E_2_ (PGE2), and RANKL are known inflammatory mediators causing alveolar bone destruction and osteoclast formation. These mediators modify the innate and adaptive immune responses that are detected immediately at infected periodontal sites.^5^ The findings of this study demonstrated that following treatment, the expression levels of inflammatory mediators (RANKL, TNF-α) decreased in the periodontal tissues in which periodontitis was induced by injection of *P. gingivalis*. However, expression of the anti-inflammatory cytokine IL-10 was increased.

The average expression of TNF-α, and RANKL in all treatment groups with nano-emulsion and mangosteen rind extract, blank and doxycycline mucoadhesive gingival patch showed a gradual decrease after topical application on days 3, 5, and 7. Overall topical application of nano-emulsion mangosteen extract showed the lowest TNF-α, and RANKL expression compared to mangosteen extract, blank, and doxycycline mucoadhesive gingival patch with a significant difference (p < 0.001). These results suggest that mangosteen nano-emulsion may alleviate the inflammatory state of rats by reducing the expression of TNF-α at the basic level. These findings are in line with previous research carried out by Bumrungpert et al. (2010), which showed that α-mangostin can inhibit the secretion of TNF-α and PGEβ.^44^

Kresnoadi et al. (2017) showed that mangosteen was significantly effective in decreasing nuclear factor kappa B (NF-κB) and RANKL expression, which can be used as an anti-inflammatory agent.^45^ RANKL is an essential mediator of the development of osteoclasts. RANKL binding with the RANK receptor on pre-osteoblast surfaces triggers the Jun terminal kinase and NF-κB, resulting in the formation of osteoclasts. In osteoimmunology, RANKL plays a significant role as well.^46^ Moreover, a previous study in U937 macrophage-like cells revealed that the release of inflammatory markers IL-4 and TNF-α can be inhibited by 20 μg/mL α-mangostin.^19^ The study also showed that mangosteen greatly affected the immune milieu within periodontal tissue.

To further investigate its effects on treating periodontitis, we examined the changes that occurred after treatment application. In this study, there was a distinct difference in the elevated level of IL-10 expression in treatment group II compared to treatment group I and the antibiotic control group. According to this study, the application of mangosteen nano-emulsion mucoadhesive gingival patch to infected rats was effective in elevating IL-10 expression. This is consistent with a study by Zhang et al. (2014), who states that mangosteen act as a subsequent suppression for the inflammatory phase.^47^ IL-10 is a robust anti-inflammatory cytokine that inhibits the immune system's proliferative and inflammatory responses, and is a factor produced by Th2 cells. IL-10 prevents Th1 cells from producing cytokines. IL-10 has additional stimulatory effects on thymocytes, B cells, and mast cells. Many other cell types including mast cells, macrophages, eosinophils, dendritic cells, B cells, and a wide variety of T cell subsets produce IL-10. Thus, IL-10 can reduce the production of proinflammatory cytokines and chemokines such as TNF-α, IL-1, and IL-6. IL-10 also has the ability to downregulate the production of nitric oxide, collagenase, and gelatinase. Therefore, in both homeostatic and inflammatory conditions, IL-10 is regarded as a key regulator of bone homeostasis. This research is consistent with a study by Kim et al. (2017), which demonstrated the anti-inflammatory effect of α-mangostin in reducing the proinflammatory cytokine TNF-α, and increasing the anti-inflammatory cytokine IL-10.^48^ Previous clinical investigations have shown that applying a gel containing mangosteen rind extract to the gums reduces periodontal inflammation, indicating that the formulation can be used as a periodontal therapy adjunct.^49^ This is due to the biologically active component of mangosteen, which has anti-inflammatory, antibacterial, and antioxidant effects and has shown good results in reducing the depth of residual pockets, gingival index, and gingival bleeding, and improving clinical epithelial attachment.^50^

From the above results, the significant foremost effect was due to the topical use of nano-emulsion of mangosteen mucoadhesive gingival patch in the periodontal tissue. The encapsulation of mangosteen extract in nano-emulsion was chosen because nano-emulsion is an advanced method to apply drugs and antimicrobial agents due to the deep effect caused by nanodroplets. Nano-emulsions are nano-sized emulsions that are constructed to improve the delivery of active pharmaceutical substances due to the nano-scaled droplet size providing a greater surface area and better absorption.^51^ In this study, the mean droplet size (diameter) of the nano-emulsion droplet (Table 1) fulfilled all characteristics of a stable homogenous nano-emulsion. A previous study reported that the mean droplet size of nano-emulsions occurs in the range of 100–500 nm^27^^,^^52^; however, other studies have shown that it ranges from 10 to 1000 nm^53^ and 50 to 1000 nm.^29^^,^^54^ Regarding zeta potential a stable nano-emulsion is formed when the zeta potential value is more negative than −30 mV or more positive than +30 mV.^52^ Whereas for PDI, a PDI value of <0.08 shows a monodisperse sample, a value of 0.08–0.7 indicates a midrange, and a value of >0.7 indicates a very broad distribution of droplet size.^55^ In a study where nano-emulsion formulation was used to treat periodontitis, the histopathological results for the rats showed a significant reduction of TNF-α, which reveals the important role of anti-inflammatory and antibacterial activity for the treatment of periodontal disease.^1^

In this study, nano-emulsion was used as a vehicle to prepare the mangosteen rind extract patch, which is considered to have potent effects with intimate contact and longer time in periodontitis cases through specific interfacial forces known as mucoadhesion, cohesiveness, and compressibility. These results suggest that the mangosteen nano-emulsion mucoadhesive gingival patch could be useful in preventing and treating periodontitis.

The limitations of this study were that due to requiring longer contact of 1 h between the mucoadhesive gingival patch and gingiva, an anesthetic with a long duration of action must be given. Because the patch slides easily from the gingival incisive sulcus, it needs to be held or tied with a ligature. Meanwhile, the results of this study confirm that nano-emulsion of mangosteen rind extract in a mucoadhesive gingival patch plays a role in periodontitis, where TNF-α, IL-10, and RANKL expression are the main indicators of periodontitis. To strengthen the results of this study, more research is needed to determine the effect of nano-emulsion of mangosteen rind extract in a mucoadhesive gingival patch on other markers of periodontitis such as OPG and RANK, and further investigation of other subspecies of mangosteen rind extracts is needed to evaluate the antibacterial activity against periodontopathic bacteria and further application in other products like gels, mouthwashes, and toothpaste that can be formed by using nano-emulsion of mangosteen extract.

## Conclusion

This study showed that topical application of a mucosal adhesive gingival patch loaded with nano-emulsion of mangosteen rind extract has therapeutic potential in periodontitis by decreasing the expression of TNF-α and RANKL and increasing IL-10 expression.

## Source of funding

This study was supported by the 10.13039/501100002385Ministry of Higher Education, Republic of Indonesia in Scheme Penelitian Disertasi Doktor (PDD) 2021 (Grant No. 275/UN3/2021).

## Conflict of interest

No conflict of interest.

## Ethical approval

The study was performed in strict accordance with the Guide for the Care and Use of Laboratory Animals, National Health Research and Development Ethics Standard and Guidelines Council (2017), Minister of Health, Republic of Indonesia. The research study obtained ethical approval (No. 756/HRECC.FODM/XII/2019, Approval Date: 4 December 2019) from the Research Ethics Commission of the Faculty of Dentistry, Airlangga University, Surabaya.

## Authors contributions

MA, HRQ, and DML carried out the research and collected the data. RDR designed and supervised the study, visualized and validated the data, acquired funding, and reviewed draft material. The data were organized, analyzed, and interpreted by HSB, who also reviewed the article. BA organized, analyzed, and interpreted the data and revised the article. All authors have critically reviewed and approved the final draft and are responsible for the content and similarity index of the manuscript.
